# Chondroprotective Effects of Combination Therapy of Acupotomy and Human Adipose Mesenchymal Stem Cells in Knee Osteoarthritis Rabbits via the GSK3β-Cyclin D1-CDK4/CDK6 Signaling Pathway

**DOI:** 10.14336/AD.2019.1104

**Published:** 2020-10-01

**Authors:** Xingyan An, Tong Wang, Wei Zhang, Hongliang Yu, Robert Chunhua Zhao, Yan Guo, Chunjiu Wang, Luxue Qin, Changqing Guo

**Affiliations:** ^1^School of Acupuncture-Moxibustion and Tuina, Beijing University of Chinese Medicine, Beijing, China.; ^2^Institute of Basic Medical Sciences Chinese Academy of Medical Sciences, School of Basic Medicine Peking Union Medical College, Peking Union Medical College Hospital, Center of Excellence in Tissue Engineering Chinese Academy of Medical Sciences, Beijing Key Laboratory, Beijing, China.; ^3^Acupuncture and Moxibustion Department, Beijing Traditional Chinese Medicine Hospital Affiliated to Capital Medical University, Beijing, China.

**Keywords:** KOA, ASCs, acupotomy, chondrocytes, proliferation

## Abstract

Adipose-derived stem cells (ASCs) are highly chondrogenic and can be used to treat knee osteoarthritis (KOA) by alleviating cartilage defects. Acupotomy, a biomechanical therapy guided by traditional Chinese medicine theory, alleviates cartilage degradation and is widely used in the clinic to treat KOA by correcting abnormal mechanics. However, whether combining acupotomy with ASCs will reverse cartilage degeneration by promoting chondrocyte proliferation in KOA rabbits is unknown. The present study aimed to investigate the effects of combination therapy of acupotomy and ASCs on chondrocyte proliferation and to determine the underlying mechanism in rabbits with KOA induced by knee joint immobilization for 6 weeks. After KOA modeling, five groups of rabbits (acupotomy, ASCs, acupotomy + ASCs, model and control groups) received the indicated intervention for 4 weeks. The combination therapy significantly restored the KOA-induced decrease in passive range of motion (PROM) in the knee joint and reduced the elevated serum level of cartilage oligomeric matrix protein (COMP), a marker for cartilage degeneration. Furthermore, magnetic resonance imaging (MRI) and scanning electron microscopy (SEM) images showed that the combination therapy inhibited cartilage injury. The combination therapy also significantly blocked increases in the mRNA and protein expression of glycogen synthase kinase-3β (GSK3β) and decreases in the mRNA and protein expression of cyclin D1/CDK4 and cyclin D1/CDK6 in cartilage. These findings indicated that the combination therapy mitigated knee joint immobility, promoted chondrocyte proliferation and alleviated cartilage degeneration in KOA rabbits, and these effects may be mediated by specifically regulating the GSK3β-cyclin D1-CDK4/CDK6 pathway.

Knee osteoarthritis (KOA) is one of the most common chronic diseases of the knee joint and is strongly linked to aging. Approximately 80% of the population over 65 years old shows radiographic evidence of osteoarthritis (OA) [1,2]. KOA is characterized by cartilage degeneration. Chondrocytes are the only cells that exist in cartilage and are highly differentiated, rarely undergo cell division [3], and have poor proliferation and migration abilities [4,5] under physiological conditions. Coupled with the lack of vascular supply, the cartilage ultimately shows a limited ability to repair itself [4]. Accumulating evidence suggests that the proliferation of chondrocytes is decreased while apoptosis is increased in OA [6-8], leading to a deficient self-healing ability and cell cycle arrest [9]. However, promoting the proliferation of chondrocytes contributes to alleviating OA cartilage degeneration [6,7,10,11,12,13]. Targeting chondrocyte proliferation provides great potential for preventing and treating KOA.

The nonsurgical management guidelines have suggested that biomechanical intervention is an appropriate treatment modality for all KOA individuals [14]. Knee joint mechanical imbalance aggravates cartilage injury, which induces KOA [15,16]. Evidence has shown that the effect of correcting inflammation by placebo injection was transient, while correcting the abnormal mechanics in the knee joint was lasting [17]. Acupotomy therapy is a biomechanical intervention based on modern anatomy under the guidance of a theory from traditional Chinese medicine on the meridian sinew. Acupotome, characterized by acupoint stimulation and local loosening, combines the advantages of an acupuncture needle and a scalpel. In a previous study [18,19], acupotomy therapy relieved cartilage injury by activating the FAK-PI3K-AKT signaling pathway mediated by integrin β1 and promoted the anabolism of the cartilage matrix by upregulating the expression of collagen II and aggrecan and downregulating the expression of MMP-3 in KOA cartilage. However, relatively little is known about the effect of acupotomy on chondrocyte proliferation. Based on the regenerative capacity of stem cells, emerging effective methods for OA treatment have been proposed for cartilage repair [20]. Stem cells have been indicated to promote proliferation [21] and increase the viability [22] of damaged chondrocytes. Because chondrocyte proliferation has a significant effect on relieving the degeneration of KOA cartilage, it is hypothesized that the combination therapy of acupotomy and adipose-derived stem cells (ASCs) has a positive effect on chondrocyte proliferation in KOA rabbits.

Chondrocyte proliferation mainly depends on the regulation of the cell cycle, which is divided into four phases: G0/G1, S, G2 and M [23]. Due to the accumulation of cyclins (CCNs) and cyclin-dependent kinase (CDK) complexes and the expression of CCNs, the cell cycle progresses sequentially and precisely [24]. The activity of CDKs is activated by binding to CCNs [25]. The binding of cyclin D and cyclin E to CDK4/6 [25] and CDK2 [26], respectively, forms the complexes as the crucial cell cycle regulators control the progression from G1 to S phase, a step that determines the initiation and completion of DNA replication [27]. Moreover, the cyclin D1/CDK4 complex phosphorylates retinoblastoma (Rb) and inhibits its activity, activating the transcription factor E2F to drive the cell cycle into the S phase. Hypophosphorylated Rb blocks the release of E2F, leading to cell cycle arrest in the G1 phase [28,29]. Cyclin D1 is highly expressed in the G1 phase and peaks at the boundary of the G1/S phase in the normal cell cycle [30]. Downregulating the expression of cyclin D1, which is the crucial cell cycle regulator for G1 to S phase, induces cell cycle arrest [31].

The phosphoinositide 3-kinase (PI3K)/Akt pathway contributes to cell proliferation by regulating the cell cycle progression of the G1/S phase [32]. Evidence shows that blocking the PI3K/Akt pathway in intracellular signaling cascades leads to the dysregulation of proliferation and apoptosis [33]. Akt regulates the cell cycle via targeted modulation of GSK3β, which is the substrate of Akt and highly correlates with cell proliferation [34]. GSK3β suppresses the activity of cyclin D1 by phosphorylation, which induces the ubiquitination and proteolysis of cyclin D1 [35]. Phosphorylation causes nuclear export leading to the degradation of cyclin D1 within the cytoplasm [36]. However, Akt inactivates GSK3β by phosphorylation, which initiates the transcription of cyclin D1, positively regulating the G1/S phase and accelerating the process of cell proliferation [37,38]. Therefore, the GSK3β-cyclin D1-CDK4/CDK6 signaling pathway appears to be crucial for regulating chondrocyte proliferation.

In this work, the data demonstrated that the combination therapy of acupotomy and ASCs promoted chondrocyte proliferation and relieved KOA cartilage degeneration, which was mediated by the GSK3β-cyclin D1-CDK4/CDK6 signaling pathway.

## Materials and methods

### Animals

All animal studies were approved by the animal care and use committee of Peking Union Medical College (Beijing, China). Thirty-five male New Zealand white rabbits (6 months old) weighing approximately 2.5 kg were purchased from the Laboratory Animal Center of Beijing Keyu Technology Co., Ltd. (Beijing, China). Rabbits were acclimatized for one week before the experiment began and were given unlimited access to food and water. All animals were raised in separate cages, with the temperature and humidity maintained at (22±2)° C and 50%-60%, respectively. Animal experiments were strictly conducted in accordance with the Guidance Suggestions for the Care and Use of Laboratory Animals (2006) by the Ministry of Science and Technology of China [39].

### Isolation and culture of human ASCs

Human ASCs were obtained from the Department of Orthopaedics of Peking Union Medical College Hospital (Beijing, China), and all procedures were approved by the Ethics Committee of the Chinese Academy of Medical Sciences and Peking Union Medical College (Beijing, China). Briefly, stem cells were isolated from human adipose tissue and cultured in Dulbecco’s modified Eagle’s medium (DMEM)/F-12 supplemented with 2% fetal bovine serum (FBS; Gibco, USA), 1× insulin-transferrin-selenium (ITS; Gibco, USA), 10 ng/mL epidermal growth factor (EGF; PeproTech, USA), 10 ng/mL platelet-derived growth factor (PDGF; PeproTech, USA), 50 μm β-mercaptoethanol (Sigma, USA), 2 mm L-glutamine (Invitrogen, USA), 100 U/mL penicillin and 100 μg/mL streptomycin. ASCs were maintained at 37°C in a 5% CO_2_ incubator with saturated humidity. ASCs were identified and cultured until mature, and all available ASCs were collected for the injection therapy.

### Induction of KOA and interventions

The rabbits were randomly divided into five groups as follows: blank control group with vehicle treatment (control group, n=7), KOA with vehicle treatment (model group, n=7), KOA treated with acupotomy (acupotomy group, n=7), KOA treated with human ASCs injection (ASCs group, n=7), and KOA treated with the combination therapy of acupotomy and human ASCs injection (acupotomy + ASCs group, n=7).The modified Videman method was performed to induce the KOA model. Before the operation, each rabbit was fasted and deprived of water for 10 to 16 hours; then, the rabbits were anesthetized by intravenous injection of 3% sodium pentobarbital (30 mg/kg) into the ear marginal vein. After anesthesia was complete, each rabbit was posited supinely on the operating table with the left hind leg exposed. The left hind leg was fixed with the resin bandage (5#: 150 mm × 1800 mm, Hengshui Kangjie Medical Instrument Co., Ltd.) from the groin to the toe to maintain the extended position of the knee joint, and additional double layers of orthopedic casting tapes (Connect Cast 160 series tape: KNT02, 5.0 cm × 360 cm, Suzhou Connect Medical Technology Co., Ltd.) were wrapped to reinforce the fixation; the antibiting bandage was then applied at the outermost layer. The toes were exposed to evaluate the blood supply and swelling. During the immobilization period, the extremity swelling and mould shedding were observed at any time, adjusting and refixing as necessary. The KOA model was established after effective immobilization for 6 weeks, and the bandages were removed. Rabbits in the control group did not undergo any operation.

**Figure 1.**
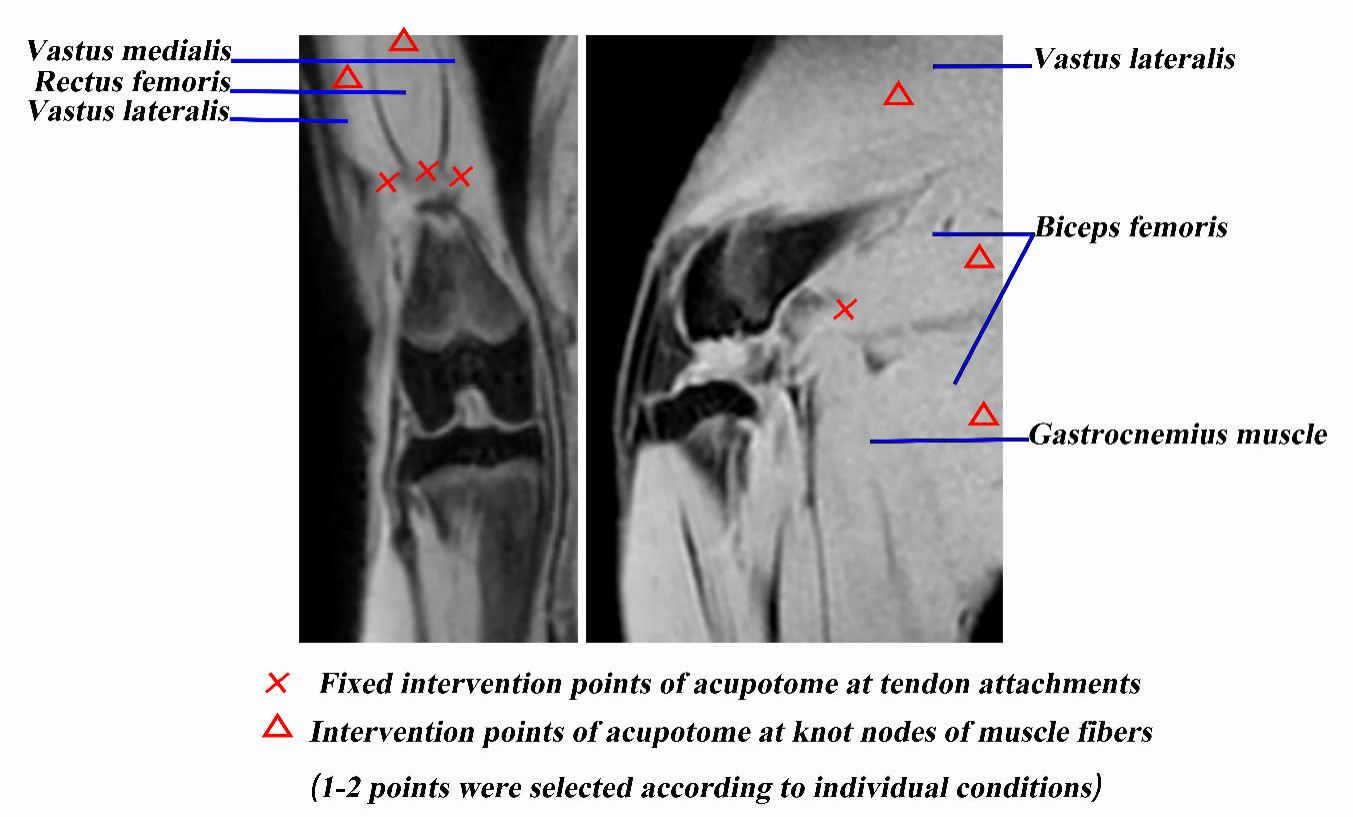
The intervention points of the acupotome.

Acupotomy intervention was performed one week after removing the bandages in the acupotomy and acupotomy + ASCs groups and was repeated twice a week for four weeks. We selected four points as the fixed intervention points, located at the tendons of vastus medialis, vastus lateralis, rectus femoris and biceps femoris (Fig. 1). In addition, 1-2 knot nodes of muscle fibers in the extensor and flexor groups around the knee joint were selected according to individual conditions (Fig. 1). After routine disinfection, the acupotome (HZ series disposable acupotome: 0.4×40 mm, Beijing Outstanding Huayou Medical Instrument Co., Ltd.) was pierced to release these points. The specific operations on each point were as follows: (1) The acupotome was inserted into the tendons by vertical insertion into the skin, and the blade of the acupotome was parallel to the longitudinal axis of the tendons. The local adhesion to the direction of tendons and bone connection was released by longitudinal dredging and transverse stripping, with 1-2 strikes per point; then, the point had been pressed for a while after the acupotome was withdrawn. (2) The acupotome was inserted into the knot nodes of muscle fibers by searching and locating the knot nodes of muscle fibers around the knee joint by palpation. The acupotome was inserted into the skin vertically, and the blade of the acupotome was parallel to the longitudinal axis of the muscle fibers. The local adhesion was released by longitudinal dredging and transverse stripping, with 1-2 strikes per point; then, the point had been pressed for a while after the acupotome was withdrawn. At the 1st week of intervention, ASCs were administered by intravenous injection at a dosage of one million ASCs dissolved in 2 ml physiological saline into the ear marginal vein of each rabbit during the first acupotomy intervention in the ASCs and acupotomy + ASCs groups. Then, during the first acupotomy intervention at the 2nd to 4th week, one hundred thousand ASCs dissolved in 2 ml physiological saline were injected into the articular cavity of the affected limb of each rabbit in the ASCs and acupotomy + ASCs groups. In the control and model groups, only physiological saline infusions of equal volumes were administered. Animals were humanely sacrificed after the indicated treatment for 4 weeks.

### Ethology evaluation

The ethology evaluation of KOA rabbits was performed one week after removing the bandages and one week after treatment, including the assessments of knee joint mobility by measuring PROM. The specific operation was as follows: the maximum extension angle and the maximum flexion angle of knee joints were measured by a digital protractor (size: SL1610A0051, Shanghai Sanliang instrument Co., Ltd.), and the difference was the value of PROM. The results were evaluated and scored separately by two independent researchers. The average scores were used for analysis.

### Magnetic resonance imaging (MRI)

The MRI examination was carried out one week after treatment. The operation of anesthesia and the preparation before anesthesia were described above. After anesthesia, each rabbit was posited supinely in the cabin with the leg at a straight position. The knee joint of each rabbit was scanned using the MRI scanner (Philips 3.0 T Achieva TX) equipped with an 8-Channel Phased Array Knee Coil (Invivo, Gainesville, FL). The scanning position was coronal, sagittal and axial with T1W 3D THRIVE and T2W 3D WISTER. The specifications were as follows: (1) T1W 3D THRIVE: field of view (FOV) 120 mm × 120 mm, slice 50, thickness 1.2 mm, matrix 200 × 204, time of repetition (TR)/time of echo (TE)/flip angle (FA) 6.8/3.4 ms / 7°, 3D FFE sequence, scan time 1’29’. (2) T2W 3D WISTER: FOV 120 mm×120 mm, slice 50, thickness 0.8 mm, matrix 252 × 256, TR / TE / FA 2600 / 200 ms / 90°, 3D SE sequence, scan time 3’36’’. The MRI images were independently evaluated by two senior radiologists in single-blind conditions.

### Scanning electron microscopy (SEM) observation

One week after treatment, the animals were sacrificed by anesthesia in an excess of pentobarbital, and the cartilage sections of the central weight-bearing area of the medial femoral condyle were fixed in 2.5% glutaraldehyde (SPI, USA) for 2 hours at 4?. The samples were washed with 0.1 M sodium dimethyl arsenate buffer, postfixed in 1% OsO4 buffer (SPI, USA) for 2 hours, dehydrated in a graded series of ethanol, dried using the carbon dioxide (Yanglilai Chemical Gas Co., Ltd, Beijing, China) critical point method to adhere the samples to the carrier and coated with gold using a sputter procedure. Images were obtained using SEM (Quanta 250, FEI, Czech Republic).

### Serous cartilage oligomeric matrix protein (COMP) assays

One week after treatment, to measure the concentration of COMP in the serum, 5 ml of ear vein blood samples were collected from each rabbit, which were fasted for 10 hours before sampling. The blood samples were centrifuged at 12,000 rpm for 15 minutes at 4°C. The supernatant fraction was separated and frozen at -80°C. Assays for COMP were performed using the Rabbit COMP Kits (R&D Systems, Minneapolis, MN) according to the manufacturer’s instructions. The level of the total protein in the supernatant was estimated, and the level of COMP was expressed as picograms per milligram of total protein.

### Quantitative real-time PCR

The cartilage of the tibia and femur was rapidly dissected and frozen in liquid nitrogen and stored in a -80°C refrigerator. Total RNA was extracted from the cartilage using TRIzol Reagent (Invitrogen, Grand Island, NY, USA) according to the manufacturer’s protocol. RNA was reverse transcribed into cDNA using a Reverse Transcription kit (TaKaRa, Japan) according to the manufacturer’s instructions. cDNA amplification was performed using Hieff qPCR SYBR Green Master Mix (Yeasen, Shanghai, China). The relative expression of the target gene was calculated by the 2^-ΔΔCt^method and normalized to the expression of GAPDH. The primers upstream and downstream are shown in Table 1.

**Table 1:** Primers used in this study.

Gene	Primer sequences
GSK3β	Forward: AGGAACACCAAAGGGAGC Reverse: TACTCCAGGAGACGGCTACA
cyclin D1	Forward: TCAAGTGTGACCCGGACTG Reverse: GCTTCTTCCTCCACTTCCCC
CDK4	Forward: GATGCGCCAGTTTCTAAGCG Reverse: GGCCAGCTTAACTGTCCCAT
CDK6	Forward: CGTGGAAGTTCAGACGTGGA Reverse: AAAGCCTGTCTGGGAAGAGC
GAPDH	Forward: TTCACCACCATGGAGAAGGC Reverse: CTCGTGGTTCACACCCATCA

### Western blot analysis

Total proteins were extracted from the cartilage using ultrasonication in RIPA lysis buffer containing 1% protease inhibitor cocktails (Beyotime Biotechnology, Shanghai, China), and then the protein concentrations were quantified with a bicinchoninic acid (BCA) protein assay kit (Beyotime Biotechnology, Shanghai, China). Equal quantities (50 µg) of proteins were separated by electrophoresis through 12% sodium dodecyl sulfate-polyacrylamide electrophoresis (SDS-PAGE) (PPLYGEN, Beijing, China) gels and then transferred onto 0.45 µm PVDF membranes (Millipore, USA). Subsequently, the membranes were blocked with 5% nonfat milk in Tris-buffered saline with 0.25% Tween-20 (TBST) solution (Baoman Biotechnology, Shanghai, China) for 2 hours at room temperature (RT) and then incubated with primary antibodies against GSK3β, cyclin D1 and CDK4 (Santa Cruz Biotechnology Co., Ltd., USA) on a shaker at 4°C overnight. GAPDH (Santa Cruz Biotechnology Co., Ltd., USA) was used as the internal control and was measured for protein loading. After washing in TBST, the membranes were incubated with HRP-conjugated secondary antibodies (Thermo Fisher, USA) for 1 hour at RT and were washed again in TBST. Finally, the bands were visualized using a chemiluminescence ECL reagent (Millipore, USA), and images were captured with a ChemiDoc XRS+ (Bio-Rad Laboratories, Hercules, CA, USA). The grayscale value of the protein bands was analyzed by Image-Pro Plus software 5.0 (Rockville, USA). The ratio of the target protein to the internal control was used to measure the relative protein concentrations of GSK3β, cyclin D1 and CDK4.

### Immunohistochemistry

The cartilage-subchondral bone complex samples of the central weight-bearing area of the medial femoral condyle were fixed in 4% paraformaldehyde (Boster, Wuhan, China) for 24 hours at 4?. Whole samples were decalcified in neutral 10% EDTA for 4 weeks at RT. The samples were embedded in paraffin after dehydrating with gradient alcohol and submersion with xylene and paraffin. The paraffin-embedded samples were sectioned from the sagittal position into 5 µm-thick sections. Immunohistochemical staining was performed using a mouse polymer method detection kit (ZSGB-BIO Co., Ltd., Beijing, China) according to the manufacturer’s instructions. The sections were dewaxed by xylene and rehydrated by gradient alcohol, and antigen retrieval was performed by heating the sections in a water bath at 95? in antigen retrieval buffer (10 mM sodium citrate; pH 6.0) for 15 minutes. The activity of endogenous peroxidase was quenched by incubation with 3% H2O2 at RT for 10 minutes, and the samples were blocked by incubation with 5% goat serum at 37? for 30 minutes. Then, the sections were incubated with primary antibodies against GSK3β, cyclin D1, CDK4 and CDK6 (Santa Cruz Biotechnology Co., Ltd., USA) overnight at 4?. Next, sections were washed in PBS, followed by incubation with HRP-conjugated goat anti-mouse IgG secondary antibodies (ZSGB-BIO Co., Ltd., Beijing, China) for 20 minutes at room temperature (RT). The chromogenic reaction occurred by incubating with DAB in the dark for 5-8 minutes at RT. All sections were evaluated for detecting the distribution of GSK3β, cyclin D1, CDK4 and CDK6. The stained sections were scanned under an optical microscope (Nikon Co., Ltd., Japan), and the ratio of integrated density to the area (IOD/area) was calculated to analyze the stained sections in a semiquantitative manner.

### Statistical analysis

All data were statistically analyzed using SPSS software (version 20.0, SPSS, Inc., Chicago, IL, USA) and are presented as the mean±SD. Variance analyses of the main effect and interaction effect were implemented by a general linear model. Multiple comparisons of differences between groups were achieved by the least significant difference (LSD) test, and those among multiple groups were assessed by performing one-way ANOVA. Differences were considered statistically significant at p< 0.05 and p< 0.01.

**Figure 2.**
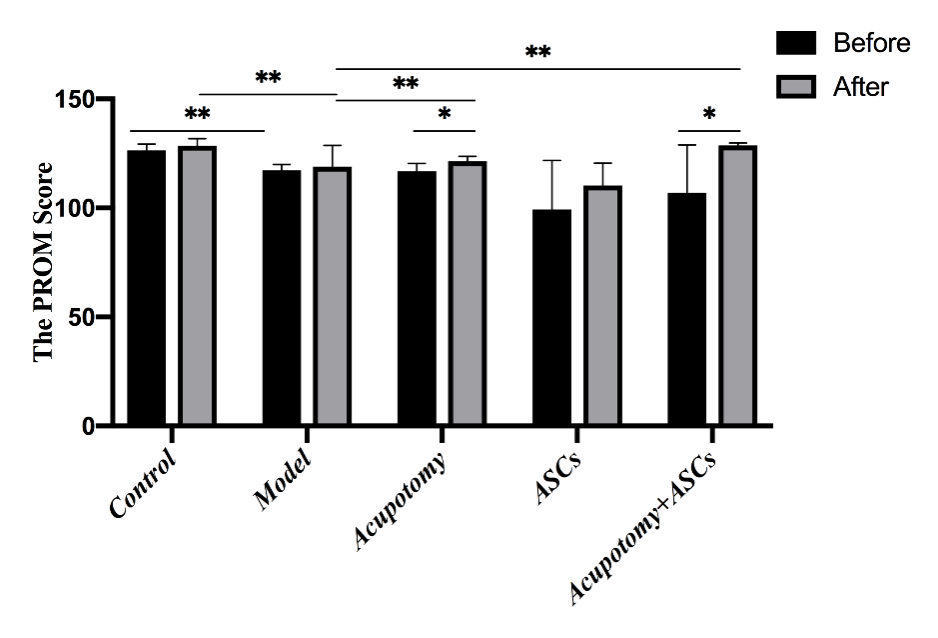
The combination therapy of acupotomy and ASCs blocked the KOA-induced decrease in the PROM score. The score analysis of PROM. Values are means ± SEMs. n = 6 per group. * p < 0.05, ** p < 0.01.

## RESULTS

### The combination therapy of acupotomy and ASCs blocked the KOA-induced decrease in the PROM score

Knee joint stiffness is one of the most typical clinical symptoms of KOA. Acupotomy has been reported to restore joint mobility. Therefore, in KOA rabbits, we evaluated the PROM score, which assessed the severity of KOA and knee joint mobility. The results analysis revealed that the PROM score was significantly decreased (p<0.01) in the KOA model group compared with that in the control group (Fig. 2). The PROM scores were significantly increased (p<0.01) in the acupotomy and acupotomy + ASCs groups compared with that in the model group after treatment, while the scores in the acupotomy and acupotomy + ASCs groups increased significantly (p<0.05) after treatment compared with that in the corresponding group before treatment (Fig. 2). There was no significant difference (p>0.05) in the PROM score between the ASCs and model groups after treatment (Fig. 2). These results indicated that there was an interaction between acupotomy and ASCs, that using acupotomy may reverse the OA-induced joint stiffness by releasing conglutinated soft tissues surrounding the knee, and that using ASCs in combination with acupotomy would yield better results.

### The combination therapy of acupotomy and ASCs relieved cartilage degeneration and inhibited the increased levels of COMP in the serum of KOA rabbits

Cartilage injury is the main pathological manifestation of KOA. Owing to the various effects of acupotomy and ASCs in treating KOA, a cartilage repair strategy based on the combination of acupotomy and ASCs has been proposed to relieve cartilage degeneration in KOA. Therefore, we examined the morphology of cartilage by MRI and SEM. Further, COMP, the protein markers in cartilage degradation, was measured in the serum by ELISA.

**Figure 3.**
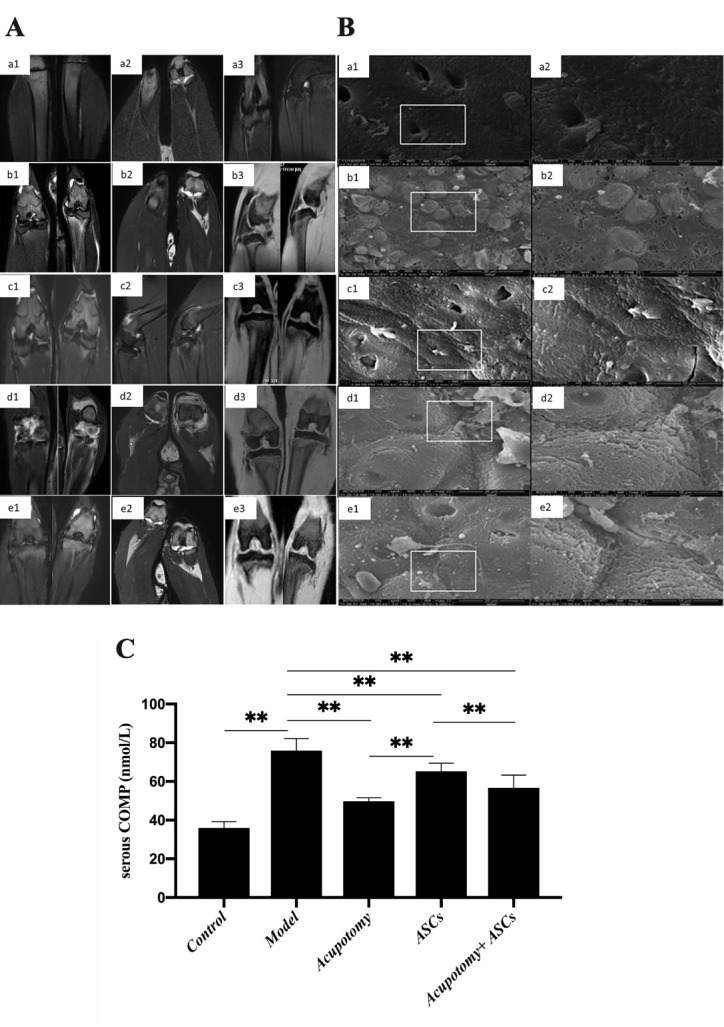
The combination therapy of acupotomy and ASCs relieved cartilage degeneration and inhibited the increased levels of COMP in the serum of KOA rabbits. (A) MRI images in the coronal position. a: control group, b: model group, c: acupotomy group, d: ASCs group, e: acupotomy + ASCs group. (B) SEM images. (Magnification: a1, b1, c1, d1, e1 are at 5000×; a2, b2, c2, d2, e2 are at 10000×). (C) ELISA of serous COMP. Values are means ± SEMs. n = 6 per group. * p < 0.05, ** p < 0.01.

The MRI images showed the entire configuration of the knee joint, including the cartilage and the perigenicular soft tissue. In the control group, the T1W 3D THRIVE sequence showed that the cartilage surface was smooth and complete, and soft tissue swelling and fascia effusion were barely observed. The T2W 3D VISTA sequence showed a small amount of effusion that was observed in the joint cavity (Fig. 3A. a1-3). In the model group, T1W 3D THRIVE sequences showed cartilage surface damage integrally, which was the typical pathological feature of KOA imaging. The T2W 3D VISTA sequence showed obvious bone edema, a large amount of effusion in the cavity, swollen soft tissues and fascia effusion (Fig. 3A. b1-3). In the acupotomy group, the T1W 3D THRIVE sequence showed that the cartilage surface was almost complete. The T2W 3D VISTA sequence showed bone edema, a small amount of effusion in the cavity, soft tissue swelling and fascia effusion (Fig. 3A. c1-3). In the ASCs group, the T1W 3D THRIVE sequence showed defects in the cartilage surface. The T2W 3D VISTA sequence showed a large amount of effusion in the cavity, soft tissue swelling and a large amount of fascia effusion (Fig. 3A. d1-3). In the acupotomy + ASCs group, the T1W 3D THRIVE sequence showed that the cartilage surface was relatively complete and rough. The T2W 3D VISTA sequence showed a medium amount of effusion in the joint cavity, and slight swelling and fascia effusion were observed (Fig. 3A. e1-3).

**Figure 4.**
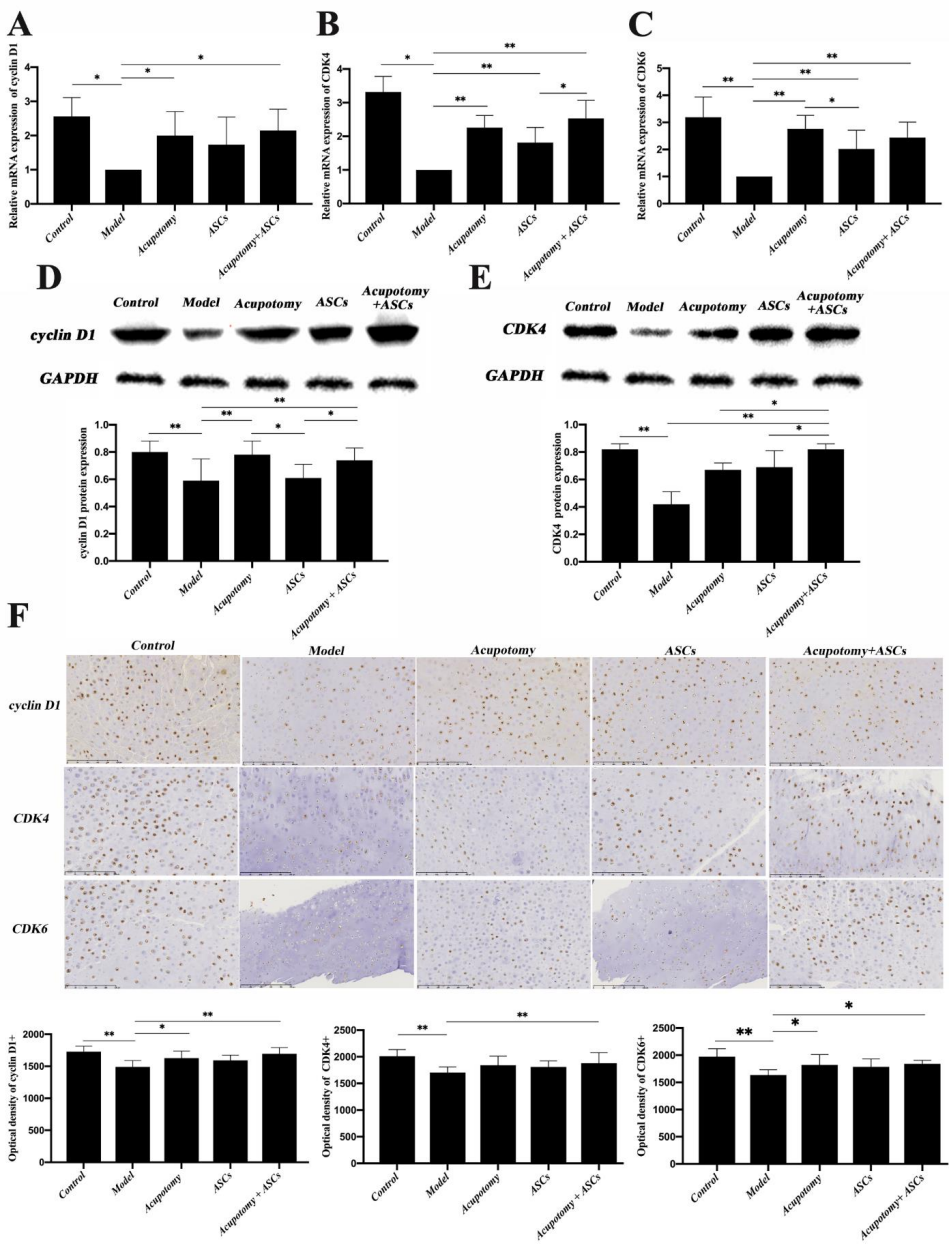
The combination therapy of acupotomy and ASCs upregulated the decreased expression of cyclin D1, CDK4 and CDK6 in the cartilage of KOA rabbits. (A) Real-time PCR analysis of cyclin D1. (B) Real-time PCR analysis of CDK4. (C) Real-time PCR analysis of CDK6. (D) Western blot assay of cyclin D1. (E) Western blot assay of CDK4. (F) Immunohistochemical staining and optical density values of cyclin D1, CDK4 and CDK6. The values are expressed as the mean ± SEMs. n = 6 per group. * p < 0.05, ** p < 0.01. Scale bars = 250 μm.

SEM observations of the morphology of cartilage demonstrated that the surface was covered with amorphous substances uniformly and that the superficial structure was basically complete in the control group (Fig. 3B. a1-2). The amorphous material covered barely visible cracks on the surface, and a large number of collagen fibers were exposed and taken up in the model group; these symptoms are the typical pathological morphology of KOA cartilage (Fig. 3B. b1-2). The surface was covered with amorphous material relatively uniformly and showed tiny cracks and less exposed collagen fibers in the acupotomy group (Fig. 3B. c1-2). In the ASCs group, the amorphous material covered the dry cracks and deep gills on the surface, and the collagen fibers were exposed and upturned (Fig. 3B. d1-2). The coverage of the amorphous material was uniform, and small cracks and exposed collagen fibers were observed occasionally in the acupotomy + ASCs group (Fig. 3B. e1-2). These results indicated that all three treatments reduced cartilage damage to some extent; however, the effect of the combination of acupotomy and ASCs on promoting cartilage repair was the most significant.

ELISA analysis revealed that the expression of the COMP protein in the serum was significantly increased in the model group (p<0.01) compared with that in the control group, while it was significantly decreased (p<0.01) in the acupotomy and acupotomy + ASCs groups compared with that in the model group (Fig. 3C). However, it was significantly decreased (p<0.01) in the acupotomy group compared with that in the acupotomy + ASCs group (Fig. 3C). The expression of COMP was significantly decreased (p<0.01) in the ASCs group compared with that in the model group (Fig. 3C). COMP expression was increased in the ASCs group compared with that in the acupotomy and acupotomy + ASCs groups (Fig. 3C). These results suggested that both acupotomy and combination therapy relieved the damage of cartilage in KOA rabbits by blocking the increase in the expression of COMP, the cartilage degradation-related marker protein.

### The combination therapy of acupotomy and ASCs upregulated the decreased expression of cyclin D1, CDK4 and CDK6 in the cartilage of KOA rabbits

Cyclin D1/CDK4/CDK6 are the crucial regulators of the cell cycle and play a significant role in regulating cell proliferation. We investigated whether the three therapies affected the proliferation of chondrocytes by regulating the expression of cyclin D1, CDK4 and CDK6. Real-time PCR analysis revealed that the expression of mRNAs in cyclin D1, CDK4 and CDK6 were all significantly decreased (p<0.05, p<0.01) in the model group compared with that in the control group, while it was significantly increased (p<0.05, p<0.01) in the acupotomy and acupotomy + ASCs groups compared with that in the model group (Fig. 4A & B & C). The comparative analysis of the three therapies showed that the mRNA expression of CDK4 and CDK6 in the ASCs group was significantly lower (p<0.05) than that in the acupotomy + ASCs group and the acupotomy group, respectively (Fig. 4B & C). The mRNA expression of cyclin D1, CDK4 and CDK6 in the acupotomy and acupotomy + ASCs groups showed no difference, but the combination therapy showed a better tendency to block the decline in cyclin D1, CDK4 and CDK6 mRNAs (Fig. 4A & B & C).

Immunohistochemistry staining yielded similar results. The protein expression of cyclin D1, CDK4 and CDK6 were all significantly decreased (p<0.01) in the model group compared with that in the control group, while the three proteins were all significantly increased (p<0.05, p<0.01) in only the acupotomy + ASCs group compared with those in the model group (Fig. 4F). The acupotomy group and the model group were compared, and only the protein expression of cyclin D1 and CDK6 was increased (p<0.05), and the expression of CDK4 showed no difference between the two groups (Fig. 4F). There was no difference between the ASCs group and the model group in the expression of cyclin D1, CDK4 and CDK6 (Fig. 4F). Compared with the acupotomy and acupotomy + ASCs groups, the combination therapy group showed a better capacity to block the decline in the cyclin D1, CDK4 and CDK6 proteins.

Western blot analysis of cyclin D1 and CDK4 further confirmed the results. The results comparing the acupotomy + ASCs group and the model group were consistent with those of real-time PCR and immunohistochemistry. The comparative analysis of the three therapies showed that the expression of cyclin D1 was significantly increased in the acupotomy + ASCs group (p<0.05) compared with that in the ASCs group (Fig. 4D), and the expression of CDK4 was significantly increased in the acupotomy + ASCs group (p<0.05) compared with that in the acupotomy group and the ASCs group (Fig. 4E). These results suggested that the effect of the combination therapy of acupotomy and ASCs on relieving cartilage degeneration may be exerted by promoting the expression of cyclin D1, CDK4 and CDK6 in the cartilage of KOA rabbits.

### The combination therapy of acupotomy and ASCs affected chondrocyte proliferation by inhibiting the activation of GSK3β in the cartilage of KOA rabbits

GSK3β, a downstream factor of the FAK/PI3K/AKT signaling pathway, affects cellular proliferation by regulating the activity of cyclin D1. A previous study showed that acupotomy relieved extracellular cartilage matrix (ECM) degeneration by activating the FAK/PI3K/AKT signaling pathway [18]. We further determined whether the therapy relieved the degeneration of KOA cartilage by promoting chondrocyte proliferation and whether GSK3β played a crucial role in blocking the decrease in cyclin D1, CDK4 and CDK6 after the homologous treatment in KOA rabbits.

**Figure 5.**
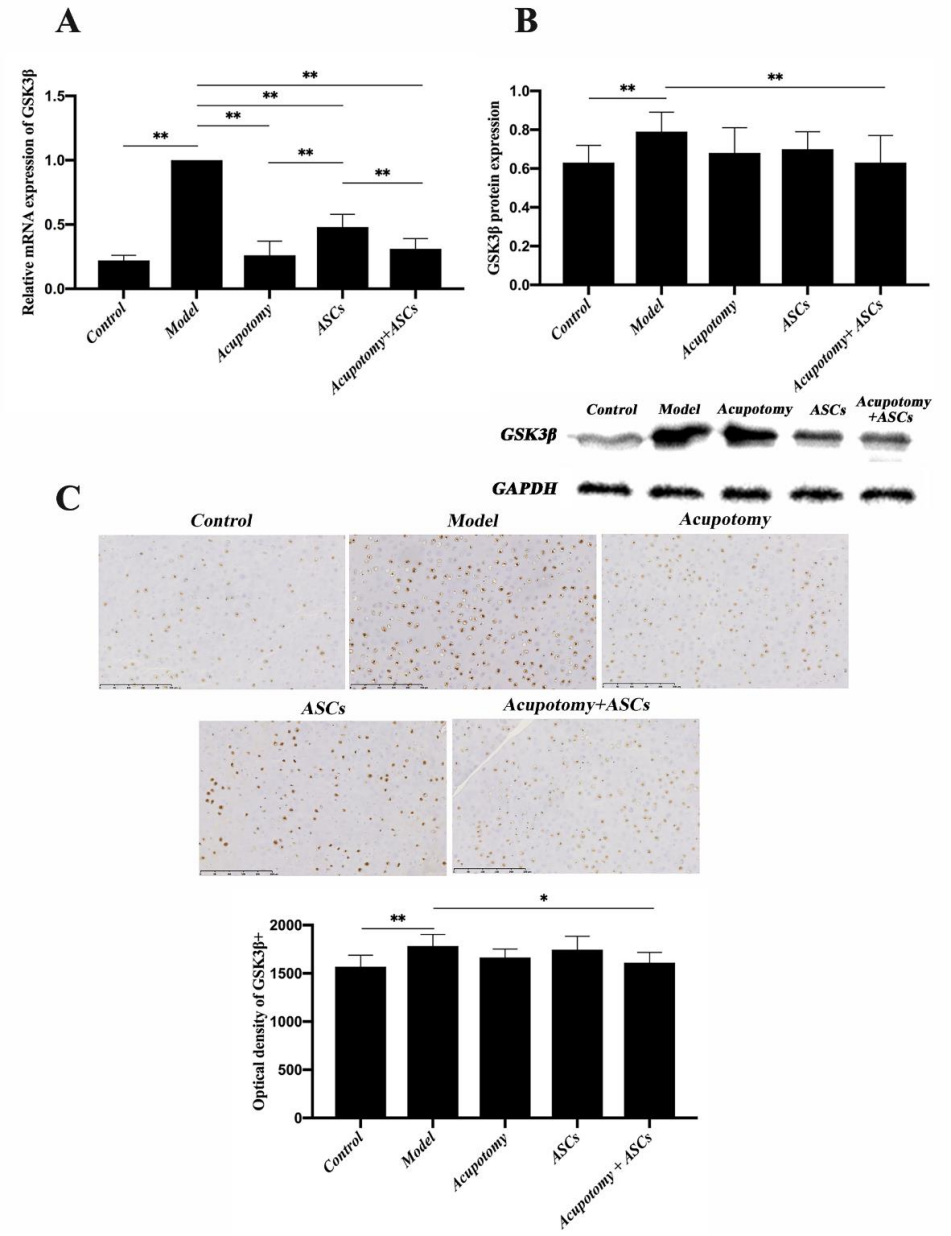
The combination therapy of acupotomy and ASCs affected chondrocyte proliferation by inhibiting the activation of GSK3β in the cartilage of KOA rabbits. (A) Real-time PCR analysis of GSK3β. (B) Western blot assay of GSK3β. (C) Immunohistochemical staining and optical density value of GSK3β. The values are expressed as the mean ± SEMs. n = 6 per group. * p < 0.05, ** p < 0.01. Scale bars = 250 μm.

Real-time PCR analysis showed that the mRNA expression of GSK3β was significantly increased in the model group (p<0.01) compared with that in the control group, while the mRNA expression was significantly downregulated in the three therapies group (p<0.01) compared with that in the model group (Fig. 5A). However, the ASCs group had a significantly higher mRNA expression of GSK3β (p<0.01) than the acupotomy group and the acupotomy + ASCs group (Fig. 5A). There was no difference between the acupotomy and the acupotomy + ASCs groups in inhibiting the increase in GSK3β mRNA (Fig. 5A). Western blot analysis confirmed that the protein expression of GSK3β was significantly increased in the model group (p<0.01) compared with that in the control group, while it was significantly decreased in only the acupotomy + ASCs group (p<0.01) compared with that in the model group (Fig. 5B). The individual therapy of acupotomy or ASCs showed little effect on downregulating the expression of GSK3β protein. Immunohistochemistry staining of GSK3β in the cartilage further verified the results (Fig. 5C). These results demonstrated that the GSK3β-cyclin D1-CDK4/CDK6 pathway might be specifically involved in the pathogenesis of KOA cartilage degradation. Further, the effect of the combination therapy of acupotomy and ASCs on relieving KOA cartilage degeneration might be exerted through promoting chondrocyte proliferation by regulating this pathway.

## DISCUSSION

In the present study, we have shown that KOA induced knee joint stiffness and articular cartilage degeneration and decreased chondrocyte proliferation. The combination therapy of acupotomy and ASCs restored the mobility of the knee joint, mitigated the erosion of cartilage and upregulated the proliferation levels of KOA chondrocytes, and these effects may be mediated by specifically regulating the GSK3β-cyclin D1-CDK4/CDK6 pathway in the cartilage of KOA rabbits. The KOA rabbit models were induced by immobilization in the knee joint for 6 weeks. We found that the combination therapy of acupotomy and ASCs can increase the PROM score of the knee joint, and the score is a direct reflection of joint function. The combination therapy can also rescue the cartilage erosion observed by MRI and SEM from the macro and micro perspective, respectively, and can suppress the increased level of cartilage COMP, the biomarker for cartilage damage. We also observed that GSK3β is specifically activated in the cartilage of KOA rabbits, which may lead to a decrease in cell cycle regulators, including the complexes of cyclin D1/CDK4 and cyclin D1/CDK6, ultimately resulting in hindering the proliferation of chondrocytes and then accelerating the degeneration of cartilage. Combination therapy can improve abnormal KOA chondrocyte proliferation by regulating the GSK3β-cyclinD1-CDK4/CDK6 pathway.

Immobilization-induced atrophy and necrosis of chondrocytes is a widely used method for KOA model induction [40]. Cartilage ECM is rich in collagen II and aggrecan [41]. The noncollagen protein COMP, the biomarker in osteoarthritis, mainly isolates from cartilage and contributes to maintaining the stability of collagen II in cartilage [42]. The changes in the concentration of COMP in cartilage, serum and urine indicate the biochemical status of KOA cartilage [43,44]. Increased concentrations of COMP indicate deterioration of cartilage degradation. Many researchers have used COMP in serum or urine as a stable marker of KOA cartilage metabolism to evaluate the degradation level of human cartilage [43,45]. In this report, we showed that rabbits immobilized in knee joints for 6 weeks induced knee joint rigidity as measured by PROM (Fig. 2), and the surface eroded integrally in cartilage as observed by MRI (Fig. 3A. b1-3). Serous COMP increased in KOA rabbits (Fig. 3C), which was consistent with the changes in serous COMP for KOA patients. According to a systematic review and meta-analysis, COMP in serum was elevated in patients with radiographic KOA [46]. Further, COMP in serum was related to the development of radiographic and painful KOA, and these results have been confirmed in a community-based cohort of middle-aged women [47]. These indicators indicated that the rabbits with their knee joints immobilized for 6 weeks can duplicate OA-like cartilage lesions similar to those observed in human clinical cases.

Due to the poor self-regeneration of chondrocytes, the cartilage injury induced by KOA is difficult to repair [48]. With the development of tissue engineering, new strategies have been proposed for the treatment of cartilage injury. The new strategies include marrow simulation-based techniques represented by micro-fracture, osteochondral transplantation represented by cartilage allograft or autograft, and cell-based techniques represented by autologous chondrocyte implantation (ACI) [49]. Proliferation [5] is an important precondition for the successful repair of cartilage injuries. However, the present clinical efficacy of cartilage injury treatment is mainly restricted by this challenge. The strategy of microfracture can encourage the formation of fibrocartilaginous repair tissue, but bone marrow mesenchymal stem cells (BMSCs) released at the fracture site may lead to hypertrophic chondrocytes and endochondral ossification [50]; for the ACI strategy, the dedifferentiation of expanded chondrocytes in vitro leads to the formation of nonfunctional fibrocartilage [51]. The therapy to maintain the proliferation of chondrocytes has been under extensive investigation. Based on the powerful regenerative ability of stem cells, systemic infusion and targeted injection have also become one of the emerging methods effective for the intervention of KOA by promoting chondrocyte proliferation.

Mesenchymal stem cells (MSCs) are characterized by pluripotency and a self-renewal ability and can be used to facilitate the regeneration of damaged tissues induced by diseases [52-54]. Recent studies have suggested that MSCs exist in every adult tissue [55], and adipose tissue, bone marrow and synovial tissue are especially rich in MSCs [56]. Adipose tissue can isolate the undifferentiated cell population characterized by a multipotent nature [57] called ASCs. ASCs have been shown to have the potential for chondrogenesis [58,59]. ASCs expanded under suitable monolayer culture conditions and eventually induced cartilage formation in 3D culture [60], which can be promoted with the assistance of growth factors, including TGF-β1 and IGF-1 [61]. Stem cells derived from the infrapatellar fat pad showed a chondrogenic response characterized by the expression of aggrecan and collagens II, IX, X and XI, suggesting that ASCs in vitro can also differentiate into cartilage-like tissue [62]. Enhancing the regeneration potential of the damaged cartilage tissue induced by KOA remains a great challenge, and stem cell injection has been found to protect against cartilage injury and delay KOA procession in the KOA animal models of mice [63] and rabbits [64]. In clinical experiments, a latest systematic review of 288 KOA patients treated with BMSCs, ASCs or peripheral blood stem cells (PBSCs) showed that stem cell therapy has a positive effect on improving the visual analog scale (VAS), Western Ontario and McMaster Universities Osteoarthritis Index (WOMAC) and radiologic evidence for KOA patients [65]. A local injection of stem cells to treat KOA can achieve long-term curative effects [66]. These results suggest that stem cell-based therapy is one of the few available treatments for KOA. The reason why we chose human ASCs for intravenous and articular injection in rabbits was that it has been proven that human ASCs can effectively alleviate KOA symptoms in clinical practice, and this study aimed to provide direct experimental evidence for the clinical treatment of KOA. In this study, we aimed to determine whether human ASCs injection can achieve chondroprotective effects by promoting chondrocyte proliferation in KOA rabbits.

As treating KOA, we tend to neglect the correction of abnormal mechanics. However, most or almost all KOA is associated with abnormal mechanics, which overwhelms all the other risk factors deteriorating the disease as KOA initiates [67]. Abnormal mechanics in the knee joint from any cause can induce the occurrence of KOA. This is why animal models of KOA are commonly induced via surgery on the knee joint, which alters mechanical loading and causes knee joint instability [40]. The KOA population exhibited muscle dysfunctions beyond that seen with aging [68]. Muscle dysfunctions mainly manifest as muscle weakness and reduced power, which are related to an increased risk of morbidity in radiographic KOA [69]. In addition, coordination of extensor and flexor muscles contributes to stabilizing the knee joint, distributing the load evenly and avoiding stress concentrating on the surface of cartilage [70]. We chose tendons around the knee as acupotomy intervention points to restore the mechanical balance of the knee joint by regulating the mechanical function of muscle tendons. Our previous study confirmed that acupotomy use the blade at the top of the needle body to loosen the attached tendons, which effectively removed the abnormal stress on cartilage and alleviated the degradation of the ECM [18,19]. The mechanical-biological effects of acupotomy have been confirmed. Restoring the normal proliferation of chondrocytes and adjusting the abnormal mechanics through combining stem cell-based regeneration techniques and biomechanical therapy may be a potential method for KOA treatment. Whether the combination of acupotomy and ASCs will reverse the degeneration of cartilage by promoting chondrocyte proliferation deserves further discussion.

In addition to cartilage degeneration, severely limited mobility in the knee joint is ubiquitous in KOA [71]. The limited mobility and cartilage erosion were already significant after 6 weeks of immobility. The evidence showed a decrease in PROM, surface cracks appeared and collagen fibers were exposed, as seen by SEM observation, and the cartilage eroded integrally, as seen by MRI scanning; these symptoms were accompanied by an obvious bone edema, a large amount of effusion in the cavity, swollen soft tissues and fascia effusion, indicating the rapid development of KOA. We found that both the acupotomy therapy and the combination therapy of acupotomy and ASCs effectively restored the mobility in the knee joint by increasing the PROM score and successfully protecting against cartilage degeneration by MRI and SEM observation. However, there was no statistically significant difference in the PROM scores between the ASCs and model groups, and MRI and SEM still showed obvious cartilage damage in the ASCs group. In addition, the increase in serous COMP was prevented by all three therapies, which was consistent with the results of previous investigations showing a positive relationship between the level of COMP and the KOA grade score [72]. Among the three treatment groups, the acupotomy group had the best inhibition effect on serous COMP increase, followed by the combination therapy, both of which were significantly better than the ASCs group. These data suggested that the promotion of chondrocyte regeneration by ASCs injection alone did not show an advantage in alleviating cartilage injury in KOA rabbits; however, the enhancement of correcting the abnormal mechanics by combining with acupotomy seemed to be more effective in slowing cartilage degeneration. Although both acupotomy therapy and combination therapy significantly slowed the progression of cartilage erosion, they did not fully reverse the pathological changes in cartilage. These data provided further support for the direct effect of acupotomy therapy and combination therapy on promoting KOA cartilage repair (Figs. 2 & 3).

What are the underlying mechanisms of the combination therapy of acupotomy and ASCs to alleviate cartilage degeneration in KOA rabbits? The present experiment has begun to be elucidated. Cyclin Ds, CDK4 and CDK6 play a crucial role in cell proliferation. Cyclin Ds assemble with CDK4 and CDK6 to form enzymatically active holoenzyme complexes [73,74]. The complexes cyclin D1/CDK4 and cyclin D1/CDK6 phosphorylate and inactivate substrates to induce expression of the target genes essential for S phase entry, resulting in propelling the progression of the cell cycle through the first gap phase (G1) restriction point into the DNA synthetic (S) phase [75]. Cyclin Ds have been widely studied for their key role in regulating cell proliferation. Amplification of the cyclin D1 gene and overexpression of cyclin D1 protein are frequently increased in many human malignancies to promote cancer cell proliferation indefinitely, while targeted restriction of the function of cyclin D1 can effectively inhibit the proliferation of cancer cells [76]. The investigations provided proof that using CDK4/6 inhibitors have shown the value of cancer therapy by governing the proliferation of breast cancer [75]. These results suggest that the regulation of cyclin D1 and CDK4/CDK6 expression is an effective strategy for governing cell proliferation. The expression of cyclin D1 in KOA cartilage has been closely investigated since the decreased expression of cyclin D1 as the grades of KOA increased [77, 78]. Studies revealed that blanking off the decrease in cyclin D1 in KOA cartilage may promote KOA chondrocyte proliferation to a normal cycle and may protect against cartilage injury [79, 80]. However, whether the combination therapy of acupotomy and ASCs can inhibit the decrease in cyclin D1 in KOA chondrocytes has not been explored. In this study, we first defined the critical role of cyclin D1 in the degeneration of cartilage in KOA rabbits. The results showed that KOA resulted in a significantly lower expression of mRNA and protein in cyclin D1 and CDK4/CDK6. It is well known that regulating the cyclin D1 protein level is one of the critical aspects in cell proliferation and tissue repair, while both acupotomy therapy and combination therapy inhibited the decrease in the complex of cyclin D1/CDK6 in KOA cartilage, and only combination therapy simultaneously upregulated the decrease in the complexes of cyclin D1/CDK4 and cyclinD1/CDK6. However, there was no statistically significant difference between the ASCs and model groups in the mRNA and protein expression of cyclin D1 and the protein expression of CDK4/CDK6. These data showed that the therapy of ASCs injection alone mainly influenced the expression of mRNA in kinases, namely, CDK4/CDK6, and did not change the level of proliferation of chondrocytes in KOA rabbits. Cooperated with the biomechanical intervention can effectively relieve the low proliferation rate of chondrocytes in KOA rabbits, which confirmed that correcting the abnormal mechanical environment of chondrocytes can be conducive to promoting proliferation for ASCs. The expression of cyclin D1 and CDK4/CDK6 was consistent with the corresponding cartilage injury, suggesting that chondroprotective effects of combination therapy may be achieved by upregulating the complexes of cell cycle regulators, cyclin D1/CDK4 and cyclin D1/CDK6, to alleviate the low proliferation state of chondrocytes in KOA rabbits and then slow down the degeneration of cartilage (Fig. 4).

It remains to be investigated how combination therapy activates cell cycle regulators. The FAK-PI3K-AKT signaling pathway is a crucial target for the treatment of OA [81]. In previous studies, we demonstrated that acupotomy can promote cartilage anabolism by specially activating the FAK-PI3K-AKT signaling pathway [18]. Akt acts as the upstream regulator of GSK3β, inactivates GSK3β by phosphorylation at Ser 9, thereby prolonging the stabilization of cyclin D1 in the nucleus [76]. GSK3β triggers the degradation of cyclin D1 by phosphorylation at the Th 286 site [35], which results in initiating the proteasomal degradation of cyclin D1. Because the GSK3β-cyclin D1 axis contributes to governing cell proliferation, researchers attempted to control cell proliferation by regulating it [82]. Induction of cyclin D1 degradation and triggered dephosphorylation of GSK3β, which caused G1 arrest and resulted in inhibiting proliferation of cancer cells [83,84]. Upregulating the expression of cyclin D1 and down-regulating the expression of GSK3β can significantly increase the proportion of chondrocytes in S phase and decrease that in G0/G1 phase. In our results in KOA cartilage, the mRNA and protein expression of GSK3β were upregulated and cyclin D1 was downregulated, suggesting that the GSK3β-cyclin D1 signaling pathway was involved in OA cartilage. We also found decreased mRNA expression of GSK3β in the acupotomy and ASCs groups, but neither acupotomy intervention nor ASCs injection alone resulted in a significant downregulation of the increased GSK3β protein. Further, among the three therapies, only the combination therapy simultaneously inhibited GSK3β and promoted cyclin D1 mRNA and protein expression in the cartilage, suggesting that the GSK3β-cyclin D1 signaling pathway may contribute to the chondroprotective effects of combination therapy (Figs. 4 & 5). The combination therapy can upregulate the cell cycle regulators via the GSK3β-cyclin D1 pathway and then restore chondrocyte proliferation in KOA cartilage, ultimately impairing long term cartilage injury (Fig. 5).

Our results demonstrated that KOA can be induced in rabbits by immobilizing the knee joint for 6 weeks. All three therapies, the acupotomy intervention, the ASCs injection and the combination therapy of acupotomy and ASCs, can improve the stiffness in the knee joint and protect against cartilage injury to varying degrees. However, the efficacy of acupotomy combined with ASCs or acupotomy alone is better than that of ASCs alone. Thus, biomechanical therapy to restore the normal mechanical environment of articular cartilage in the treatment of KOA is helpful to promote the functional recovery of chondrocytes. The effect of correcting the abnormal mechanics affecting chondrocyte proliferation is better than that of the simple cell regeneration technique. Injecting ASCs on the basis of correcting abnormal cartilage mechanics is beneficial for ASCs to play a role in promoting chondrocyte proliferation and for accelerating the recovery of KOA chondrocytes to their normal proliferation levels.

In summary, we found that the combination therapy of acupotomy and ASCs elevated the mobility in the knee joint and mitigated cartilage injury by upregulating the decreased chondrocyte proliferation levels in KOA rabbits; this upregulation may be mediated by the GSK3β-cyclin D1-CDK4/CDK6 signaling pathway. Therefore, the techniques of chondrocyte regeneration combined with the correction of an abnormal cartilage biomechanical environment may represent a new strategy to treat KOA.
